# Synthesis of trimetallic iron-boron core and gold shell nanoparticles for experimental cancer radiotherapy

**DOI:** 10.3389/fbioe.2024.1448081

**Published:** 2024-09-11

**Authors:** Brad Coward, Jiawei Wang, Boris Kysela

**Affiliations:** ^1^ Chemistry Laboratory, Chemical Engineering and Applied Science, Engineering and Physical Science, Aston University, Birmingham, United Kingdom; ^2^ Aston Medical School, College of Health and Life Sciences, Aston University, Birmingham, United Kingdom

**Keywords:** nanoparticle, core-shell, trimetallic, non-aqueous, redox-transmetalation, nanotherapeutics

## Abstract

Cancer is a significant and constantly growing clinical problem all over the word. For many types of cancer there has been little change in mortality rate of CRC in the past decades and treatment options are limited. A striking example is malignant Glioblastoma (GBM) which exhibits a high degree of infiltration of surrounding healthy brain tissue, extremely high mortality rate, morbidity and most life-years lost of any cancer. Considerable research efforts in the last several decades have failed to improve these outcomes. Boron Capture Neutron Therapy (BNCT) is an experimental radiotherapy (RT) that shows the best hope for the patients for whom all current therapies fail. BNCT involves the intracellular release of alpha and Li-ion particles from boron in response to neutron beam and therefore its success is critically dependent on achieving high intracellular concentrations of boron atoms within the cancerous cells. Boron phenylalanine (BPA) is the most used compound to deliver boron atoms, but achieving high intracellular concentration of BPA is difficult with this small molecule compound and is an absolute limiting factor for the better outcome of BNCT. Our approach focused on a delivery of a high and stable concentration of boron atoms in a form of novel trimetallic core-shell nanoparticles, combining boron for BNCT and iron for magnetic targeting in the core, and a gold shell for stability and attachment of targeting therapeutic peptides. The research was targeted towards comparing different synthesis variables to form these core-shell particles and incorporate as much boron into the core as possible via redox-transmetalation. Partial gold shells were formed around the core via island growth with a molar ratio of Fe/B of 0.64 and high incorporation of boron.

## Graphical Abstract

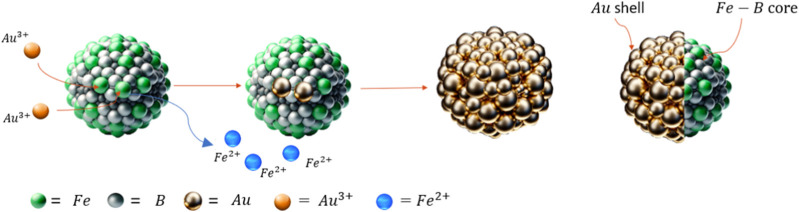

## 1 Introduction

Magnetic nanoparticles (NPs) have gained a vast amount of interest over recent years due to the range of properties that these particles gain at this scale compared to the material’s bulk properties. The uses of these magnetic nanoparticles vary from targeted drug delivery (Kianfar, 2021) to catalysis (Amiri et al., 2019) and agriculture (Singh et al., 2021). The magnetic particles transport the necessary drug to desired tissue and cells for medical applications. The advantage of using these magnetic NPs for drug delivery is that they can be controlled and manipulated via magnets to the targeted areas without requiring complicated biological targeting (Li et al., 2021). Another advantage of these magnetic NPs is that the particles are small enough to enter biological materials ranging from small cells, proteins and genes. This means these particles can be controlled with magnetic properties and enter these biological structures (Kianfar, 2021).

However, as iron is susceptible to oxidation, altering its magnetic properties, it is key to add an inert layer to protect the core from oxidation. Most commonly for medical applications, gold is used as an inert shell layer to protect a core from oxidation (Jafari et al., 2010). Another reason a gold shell is key and effective for a magnetic nanoparticles in medical applications is iron’s cytotoxicity is high leading to increased reactive oxygen species (ROS) and can cause damage to the cells (Paunovic et al., 2020). Gold answers all these problems, as a complete shell would avoid any contact from the iron core and is not cytotoxic to the cells meaning the necessary materials can be transported to the desired area safely. Although gold is typically a very inert and low cytotoxicity material used frequently in medicine; gold is susceptible to generate ROS through the activation of auger electrons. In (Mcquaid et al., 2016), a model is suggested to show the photo interaction of gold NPs that produce the characteristic high energy photoelectron and a small amount of low energy auger electrons as a result of inner shell vacancy. This can be an enhancement to the ionising dose of radiation delivered to tumours. With gold having unique optical and surface chemistry properties, it can be readily visualized and functionalized to improve the loading and targeting properties of therapeutic nanoparticles.

To turn this commonly used Fe@Au bimetallic nanoparticle into a BNCT nanotherapeutic drug, boron-10 also needs to be incorporated into the core. This specific elemental composition has several potential medical applications, the principal one being a vehicle for a targeted magnetic delivery of boron 10 to tumour cells for a potential treatment by BNCT. The BNCT is a promising experimental radiotherapy technique for the treatment-nonresponsive tumours with an extremely poor prognosis (e.g., glioblastomas), nevertheless a wider application of BNCT has been hampered by lack of methodology for a sufficient loading of tumour tissues with boron atoms—a critical prerequisite for a successful therapeutic outcome.

In theory, these trimetallic nanoparticles could be produced via redox-transmetalation. This works when the core material has a more negative reduction potential than the shell material, leading to spontaneous reduction of the shell material and oxidation of the core’s surface layer. A range of reduction potentials can be seen in Supplementary Table 1. In the case of this research gold nanoseeds form at the surface of the core. These reactions can occur spontaneously under permissible redox conditions where the electrochemical potential (ΔE) is thermodynamically favourable and occurs selectively on the metal surface. Can be an efficient formation technique for bimetallic structures (Lee et al., 2005).

The work described in this paper focuses on non-aqueous methods using the redox-transmetalation process to synthesise the trimetallic core-shell nanoparticles of iron-boron@gold. The method will be evaluated for its effectiveness in the shelling process of the gold onto the iron-boron core while investigating the impact of experimental parameters on its effectiveness. This research aims to produce a core-shell NP of Fe-B@Au with a therapeutic use for NCT.

## 2 Methodology

### 2.1 Materials

Anhydrous iron (III) chloride, sodium borohydride, chloroauric acid (HAuCl_4_. xH_2_O), 1-methyl-2-pyrrolidinone (NMP) extra dry, 4-benzylpyridine, methanol and ethanol were all purchased from Fisher Scientific and of analytical grade without further purification.

### 2.2 Experimental procedure

This method was adapted from (Corrias et al., 1993; Ban et al., 2005) for the core and shell synthesis respectively.

Initially, 2 mmol (0.324 g) of FeCl_3_ were dissolved in 50 mL of NMP to form an orange/yellow solution. Then a second solution containing 50 mL of NMP, 6 mmol (0.225 g) of NaBH_4_ was formed to produce a colourless solution. The NaBH_4_ was added rapidly to the FeCl_3_ solution and allowed to mix for 2 h to ensure the complete reaction between the two chemicals to form Fe(BH_4_)_3_ and sodium chloride (NaCl). The colour of the solution would change from orange to a reddish-orange colour. A 1:3 molar ratio of FeCl_3_:NaBH_4_ was used so that there were no excess BH_4_^−^ that may affect the redox-transmetalation process. The solution was then heated up to 100°C for 1 h which would start the hydrolysis of the Fe(BH_4_)_3_ to form the Fe-B core NPs leading to a black solution.

The Fe-B solution was then cooled down to 60°C. 0.288 g of HAuCl_4_ was added to 25 mL of NMP, resulting in a yellow solution. This gold solution was then added very slowly at a rate of 25 mL/h to the Fe-B solution. Upon the complete addition of the gold solution, the solution was heated up to 125°C for 30 min to initiate the redox-transmetalation process where the surface of the core is used as the reducing agent to reduce the Au^3+^ ions and form a uniform shell around the Fe-B core. The solution was allowed to return to room temperature and then washed several times with ethanol (EtOH) and methanol (MeOH) to remove by-products. This was followed by magnet separation to purify the sample.

### 2.3 Characterisation

Scanning electron microscopy and energy-dispersive X-ray spectroscopy (SEM/EDX) analyses were conducted using a Quattro SEM (Thermo Fisher Scientific, United States) coupled with an EDS UltraDry 60M (129 eV) detector. STEM mode was used and copper/polycarbon grids with a mesh size of 300 was used for this analysis. Dynamic light scattering (DLS) was carried out with a Nanolive Zetasizer for particle size measurement. Ultraviolet–visible spectroscopy (UV/VIS) spectrum of the nanoparticles was recorded by a UV–VIS spectrophotometer (Jenway 7615). Transmission Electron Microscopy (TEM) was carried out using an XMaxTLE TEM. X-ray photoelectron spectroscopy (XPS) was conducted in a Thermofisher ESCALAB 250 electron spectrometer. Inductively coupled plasma—optical emission spectrometry (ICP-OES) was carried out on a Thermo Fisher iCAP 7000 series ICP-OES Spectrometer.

### 2.4 Equations

$${\left(L\right)}_{n}{Fe}^{x+}+{3BH}_{4}^{-}\to {\left(L\right)}_{n}{Fe}^{x+}{\left({BH}_{4}^{-}\right)}_{x}\left(x=2\;or\;3\right)$$ (1)

## 3 Discussion

### 3.1 Fe-B core synthesis

To confirm the successful synthesis of Fe-B core, the samples were analysed by SEM, DLS and XPS for the shape, size and elemental composition. As shown in Supplementary Figure 1A, the Fe-B cores have a smooth morphology and quasi-spherical shape, indicating a uniform synthesis process. The particles are seen to form aggregates, which may be attributable to magnetic interactions. The STEM-HAADF image (Supplementary Figure 1B) shows a stark contrast between the bright and dark regions, reveals the internal structure and compositional variations within the Fe-B nanoparticles. In the SEM images, the size of the particles is estimated to be approximately 100 nm, which is corroborated by DLS measurements in Supplementary Figure 2 indicating an average hydrodynamic diameter of 137.1 ± 52.04 nm.

The incorporation level of boron within the Fe-B core structures is critical for their prospective applications. Due to carrying out the synthesis in non-aqueous conditions, the sodium borohydride does not start hydrolysing as it would do in aqueous conditions leading to immediate reduction of the iron salt. As seen in Equation 1, a complex is formed between the iron salt and borohydride and only upon heating the reduction process would occur.

As suggested previously (Corrias et al., 1993), using a higher ionic state of iron salt, Fe^3+^, should lead to the higher incorporation of boron due to three BH^4−^ being incorporated into the complex. To confirm this, the same experimental procedure was carried out with an Fe^2+^ and Fe^3+^ salts. As seen from Supplementary Table 2, using FeCl_3_ compared to FeCl_2_, there is a significant difference in the amount of boron that is incorporated into the core. When FeCl_2_ is used as the iron source, the resulting B/Fe ratio is 0.32, indicating that for each atom of iron, there is roughly one-third of an atom of boron incorporated into the nanoparticle. On the other hand, the use of FeCl_3_ results in a B/Fe ratio of 0.91, which is almost three times higher than that obtained with FeCl_2_. It substantiates the findings previously concluded by Corrias. Therefore, for the required end use of the nanoparticles, Fe^3+^ salts should be used as iron source and the experimental procedure for the core synthesis is confirmed to be an effective process.

### 3.2 Gold shell addition

To facilitate the growth of a gold shell on the core particles, the procedure exploits the reduction potential disparity between the core and the gold ions. The surface iron atoms of the core act as a reducing agent, enabling a redox-transmetallation process for gold shell formation. The literature emphasises the importance of controlled addition rates of the gold precursor solution and the initial concentration of nanoseeds in achieving a uniform gold shell (Ban et al., 2005), (Chem et al., 2012). Experimentally, two contrasting approaches were examined: a gradual addition of gold to a cooled solution (Exp A1) and a swift addition at elevated temperatures (Exp A2). The specific conditions employed and their outcomes are detailed in Supplementary Table 3.

**FIGURE 1:**
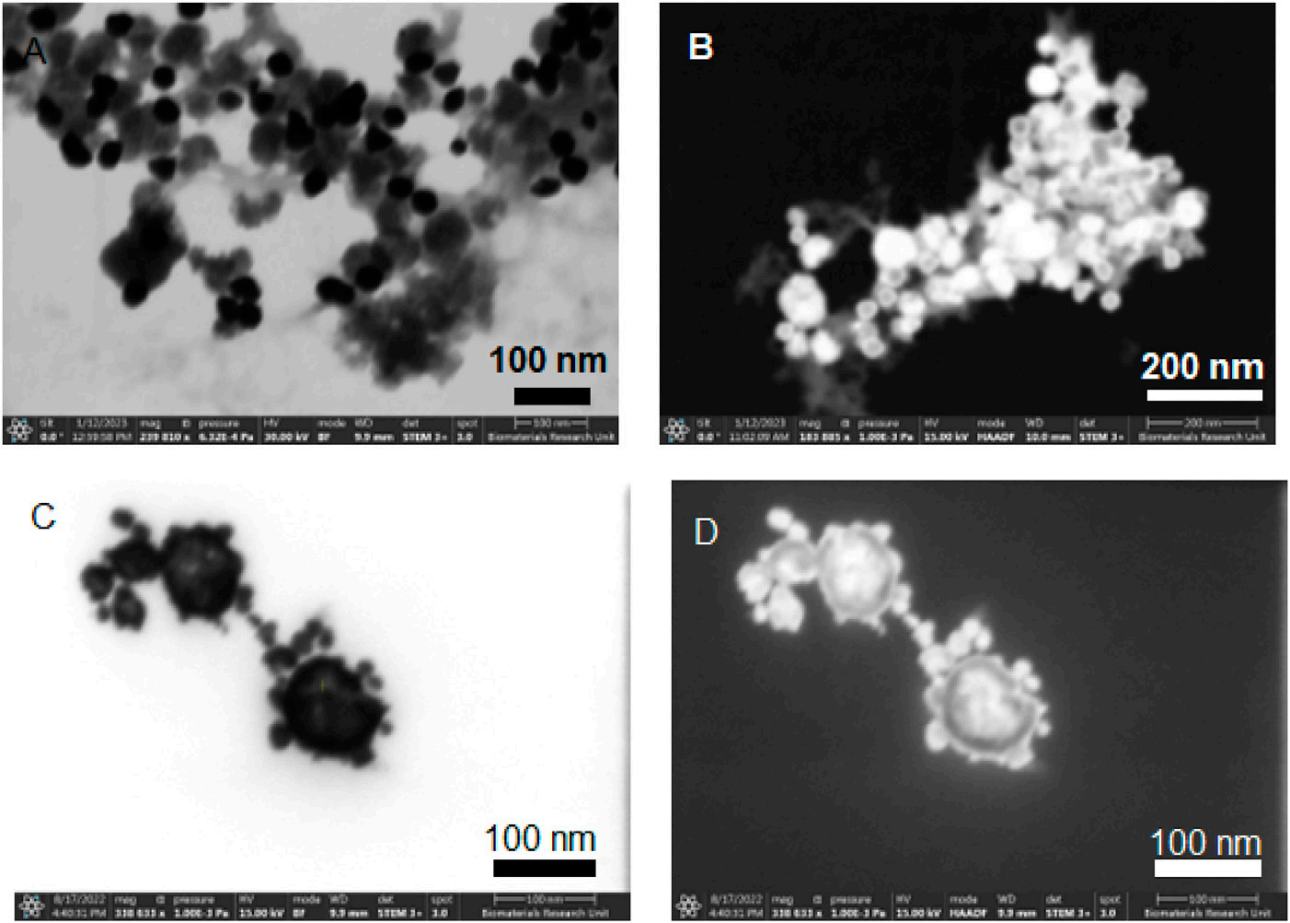
STEM images of non-aqueous experiment A1 [**(A)** STEM mode with Bright Field, **(B)** STEM mode with High Angle Annular Dark Field) and A2 **(C)** STEM mode with Bright Field, **(D)** STEM mode with High Angle Annular Dark Field].

STEM imaging was conducted on samples from experiments A1 and A2 to discern the impact of varying the addition rate and temperature of the core solution on the synthesis of nanoparticles. Figures 1A, B represent the bright field (BF) and HAADF STEM images for Exp A1, respectively. In these images, a bimodal distribution is apparent, where the amorphous, brighter entities represent the Fe-B particles, and the smaller, darker spherules are gold nanoparticles. This segregation of Fe-B and gold particles indicates that under the slower addition rate and lower temperature conditions of Exp A1, the formation of a core-shell structure was not achieved. Figures 1C, D showcase the bright field and dark field STEM images from Exp A2. The contrast between these structures is pronounced; the rapid injection of gold under a dropwise addition at a reduced temperature appears to facilitate the formation of a core-shell architecture. The images suggest a more contiguous and effective encapsulation of the Fe-B particles by gold, indicative of an improved shelling process due to the accelerated synthesis of gold nanoseeds.

**FIGURE 2:**
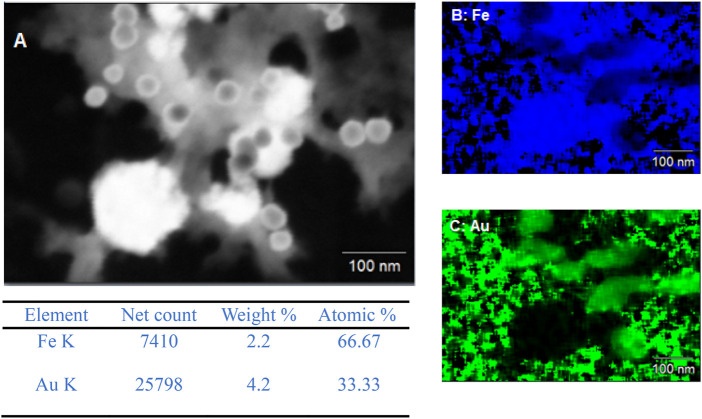
**(A)** SEM image of Exp A1 and EDX mapping of **(B)** iron and **(C)** gold.

This is further confirmed via the EDX data of Exp A1 seen in Figure 2. EDX is not a sensitive technique for light elements such as boron and so could not be picked up during EDX. However, an indication that boron is present in the core is due to the amorphous structure of the core. In (Linderoth and Morup, 1998), it is suggested that if no boron is present a crystalline iron particle is produced. However, as the boron content is increased, the particle becomes more amorphous. The absence of a core-shell structure is supported by the EDX data, where no clear overlay of iron and gold mapping would be indicative of such a structure. In this case, the cooling of the core solution and the slow addition of gold did not favour the formation of a gold shell around the iron-based core.

**FIGURE 3:**
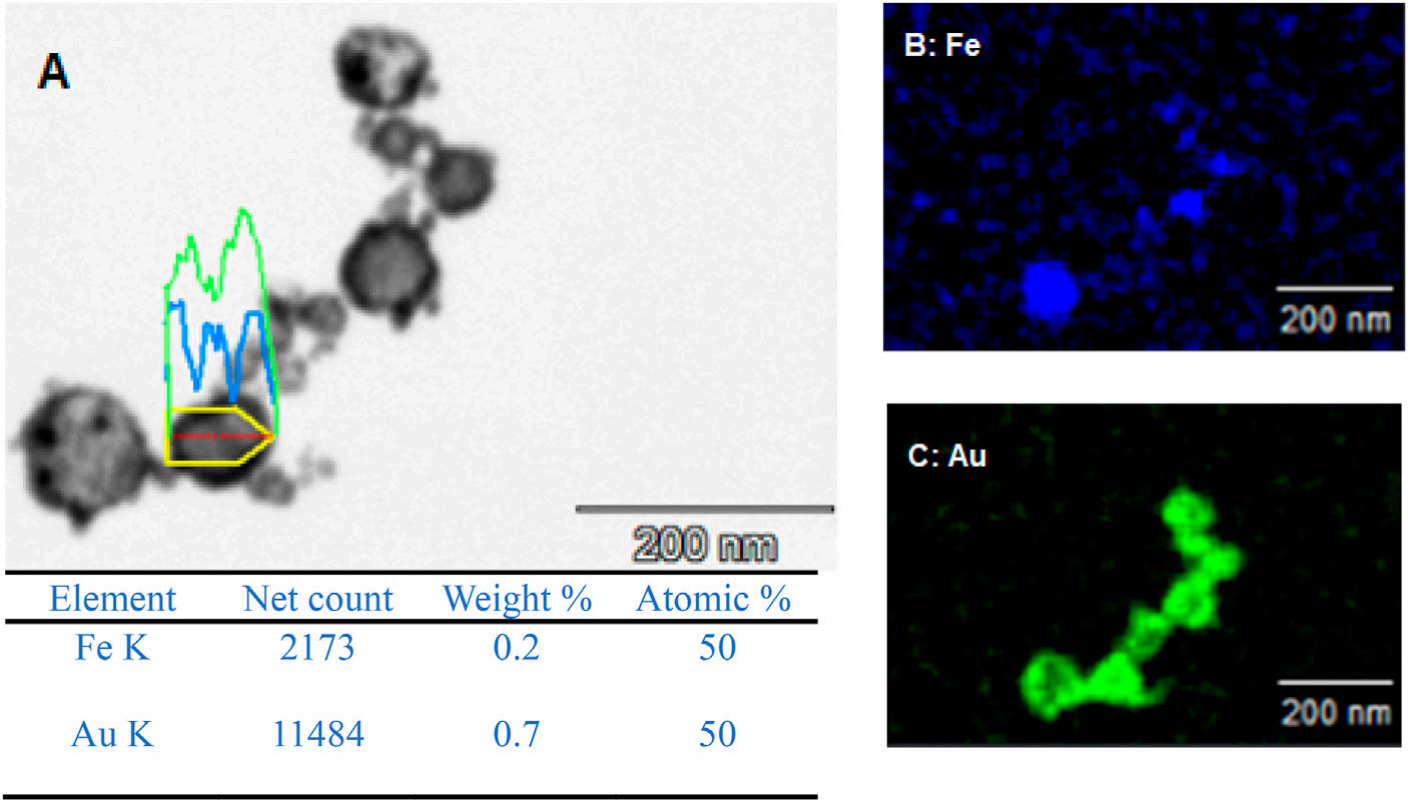
**(A)** SEM image of Exp A2 and EDX mapping of **(B)** iron and **(C)** gold.

To confirm the presence of some form of core-shell structure, EDX was carried out on the A2. As can be seen from Figure 3, there is a clear overlap between the iron detection and the gold detection suggesting a core-shell structure. This is also further backed up by the line analysis, shown in Figure 3A, carried out whereby on the edges of the particle there was a higher count rate for gold and very small for iron as expected with gold being the shell material. However, as you move through the material towards the centre, the gold counts decrease and the iron counts significantly increase with more of the core material being present in the centre of the particle. From this analysis, to give an approximate of the size of the core-shell nanoparticles, the size was measured between 100 nm and 45 nm.

The EDX data for both A1 and A2 further support that the redox-transmetalation process is occurring. In A1, the atomic % split between Fe and Au is 66.7% and 33.3% respectively, whereas in A2 the atomic % is 50:50. This displays that in a successful experiment the Fe is being used as the reducing agent for the Au ions which in turn, reduces the atomic % of Fe and increases that of Au. Furthermore, when comparing the EDX and ICP data of A2, the % split between the Fe and Au, both sets of analysis are within an agreeable limit of each other with the ICP % split between Fe and Au only is 42% and 58% respectively.

Gold NPs have very distinct optical properties that give a characteristic curve when analysed by UV-Vis. In Supplementary Figure 4, UV-Vis was carried out on both experiments A1 and A2 which was compared to a 50 nm gold colloid solution. The characteristic curve for the 50 nm gold NPs shows a peak at 540 nm. For experiment A1, where there is a segregation of Fe-B and Au NPs, a similar characteristic peak with a slight shift in wavelength to 550 nm occurs suggesting the formation of monometallic gold NPs. However, for A2, which in the STEM images displayed partial core-shell structures showed a completely contrasting UV-Vis spectra with a much broader peak with a shift to a higher wavelength. In (Ban et al., 2005), who carry out a similar synthesis process in producing Fe/Au core-shell NPs, displayed similar UV-Vis data where the Fe/Au core-shell NPs has a much broader spectra. In this case, UV-Vis could be used as a method of indication if core-shell NPs have been formed.

**FIGURE 4:**
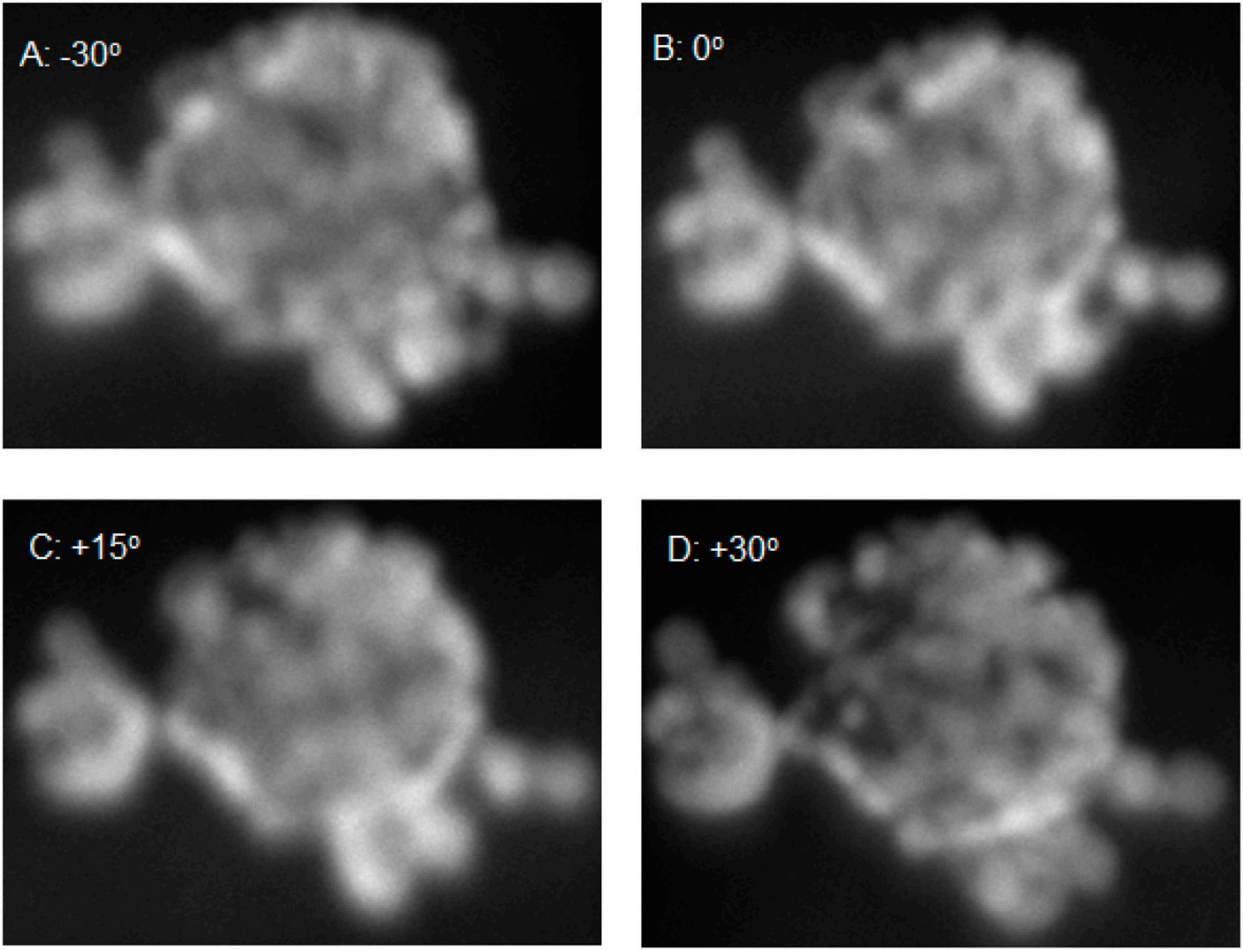
TEM images of A2 with tilt ranging from −30^o^ to +30^o^ [**(A)** −30^o^, **(B)** 0^o^, **(C)** +15^o^ and **(D)** 30^o^].

To further investigate the structure and morphology of the core-shell particles TEM was carried out at different tilt ranges to build up a 3D structure of the particle of EXP A2. From Figure 4, it is clear that there is some fore of core-shell structure but seems to be a partial shell with holes in the shell that would lead to the oxidation of the core. It appears that island growth has occurred rather than one uniform layer of gold across the core. In (Da Silva et al., 2017), it is suggested that the addition time of the shell material is vital in forming a uniform shell. In the case of Ag@Pt, if co-reduction along with redox-transmetalation occurs then island growth can start to form as the gold nanoseeds are not formed just by the core material but also residual reducing agent. However, when it is solely redox-transmetalation a more uniform shell is formed around the core. In Supplementary Figure 4, this suggests that possibly both redox-transmetalation and co-reduction occurred due to the appearance of small gold nanoparticles forming a shell rather than one uniform layer.

To compare the size of particles seen in the STEM data, DLS was carried out to further prove the size of the particles produced. Supplementary Figures 3A, B both back up the STEM data obtained that there are two distributions of particles within the solution that are the smaller monometallic gold particles and the larger amorphous iron-boron particles. Due to the product of both A1 and A2 containing a mixture of Au, Fe, B, and partially coated Fe-B@Au nanoparticles, the core particles which are uncoated have further opportunities to continue to grow and have not been capped by the gold shell leading to a wider size distribution. With the optimisation of the process, obtaining complete core-shell particles, the solution can go through an acid wash to remove all incomplete core-shell particles leaving behind the complete core-shell that would show a tighter size distribution as the gold layer would protect the inner Fe-B core. In (Ban et al., 2005), it is also suggested during the TEM analysis that monometallic Au particles are formed that have an average size of 10 nm. The method has been adapted from (Ban et al., 2005) and from Supplementary Figures 3A, B there is a smaller size distribution around 10 nm–20 nm which can be accounted for monometallic Au particles.

As A2 was the more successful experimental procedure and should be used for any further work, ICP analysis was carried out to understand the percentages of the three materials present to understand how much boron had been incorporated into the core when the complete process had been completed. From Supplementary Table 4, the ICP data shows that after a gold shell has been added there is still 1.20 M ppm of boron in the core that would be used as an effective radioactive dose of energy for the treatment of glioblastomas. When comparing Supplementary Table 2, of the XPS data, with Supplementary Table 4, of ICP data, the Fe/B ratio has decreased further from 1.10 to 0.64. This suggests that the redox-transmetalation process is successfully occurring with the reduction of gold ions by the iron surface.

## 4 Conclusion

Overall, using non aqueous methods and redox-transmetalation to try and form core-shell NPs of Fe-B@Au did not lead to a complete uniform shell of gold but a partial island growth shell when the gold was injected into the solution rather than added dropwise. However, this shows some interesting possibilities for further work and optimization around the addition time of the gold solution to the core solution. The rapid addition at higher temperatures compared to slow addition and lower temperatures leads to significant differences to the structure of the particles with rapid addition being relatively successful and slow addition unsuccessful.

The core synthesis has proven to incorporate a high amount of boron into the core with almost a one-to-one ratio before the gold shell and after a gold shell is added a 39.87% in the core. This increase in concentration from the core to the entire particle can be put down to using the iron surface as the reducing agent.

Further improvements of our strategy to form a complete shell followed by biological testing are certain to lead to intriguing therapeutic applications and further hope for cancer patients for whom all current therapies fail.

## Data Availability

The original contributions presented in the study are included in the article/Supplementary Material, further inquiries can be directed to the corresponding authors.
