# Supplementary material for: Synthesis of trimetallic iron-boron core and gold shell nanoparticles for experimental cancer radiotherapy
Source: Front Bioeng Biotechnol. 2024 Sep 11;12:1448081. doi: 10.3389/fbioe.2024.1448081 (PMC11422082; doi:10.3389/fbioe.2024.1448081)
Supplement: Supplementary file 1 [file Presentation1.pdf]

Supplementary Material

1.1 Supplementary Figures

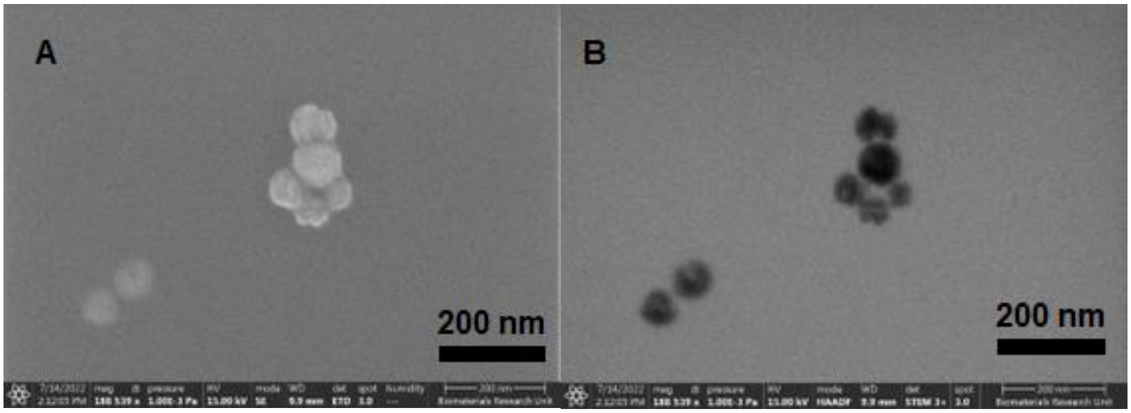

Figure S1. SEM images of the Fe-B core samples. A: Everhart-Thornley Detector (ETD) mode; B: Scanning Transmission Electron Microscopy (STEM) mode with High-Angle Annular Dark Field (HAADF)

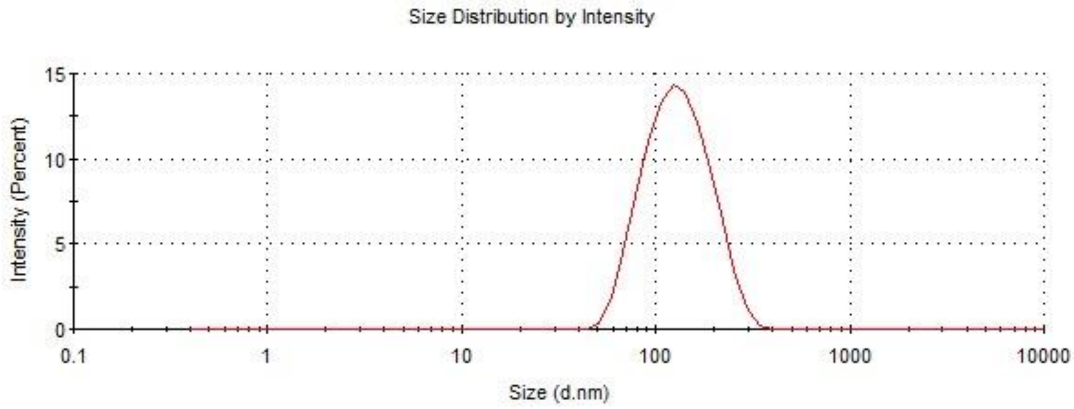

Figure S2: DLS data of the iron-boron core.

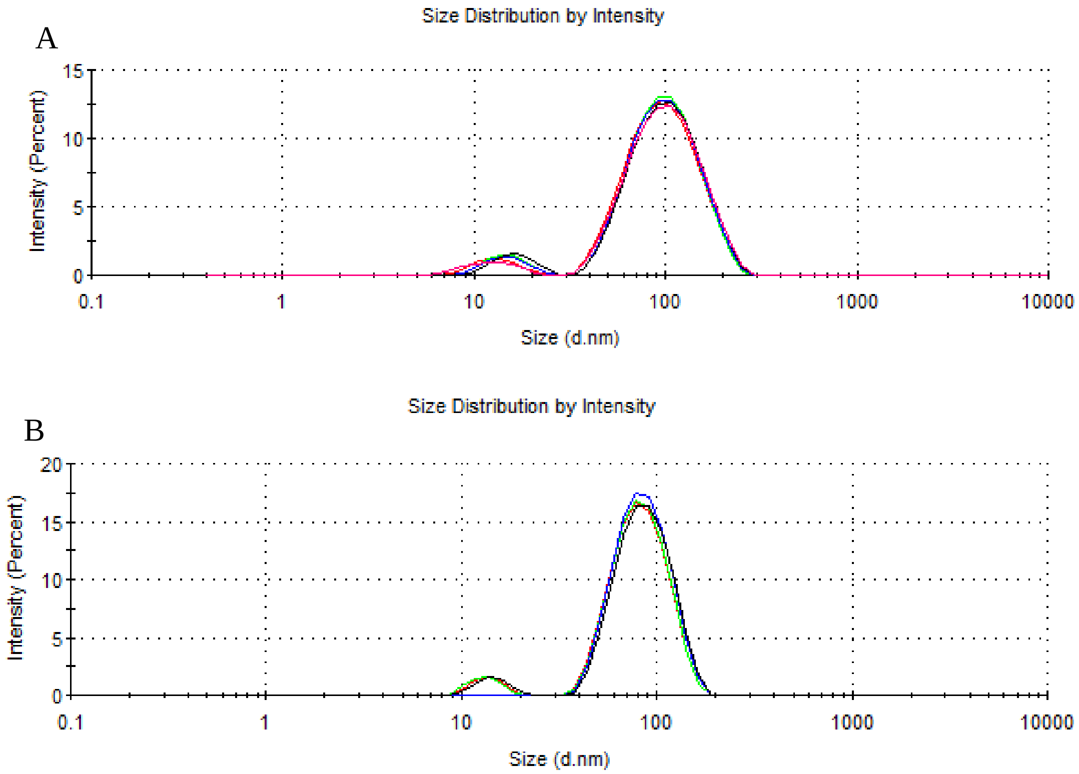

Figure S3: DLS results of A – A1 and B - A2

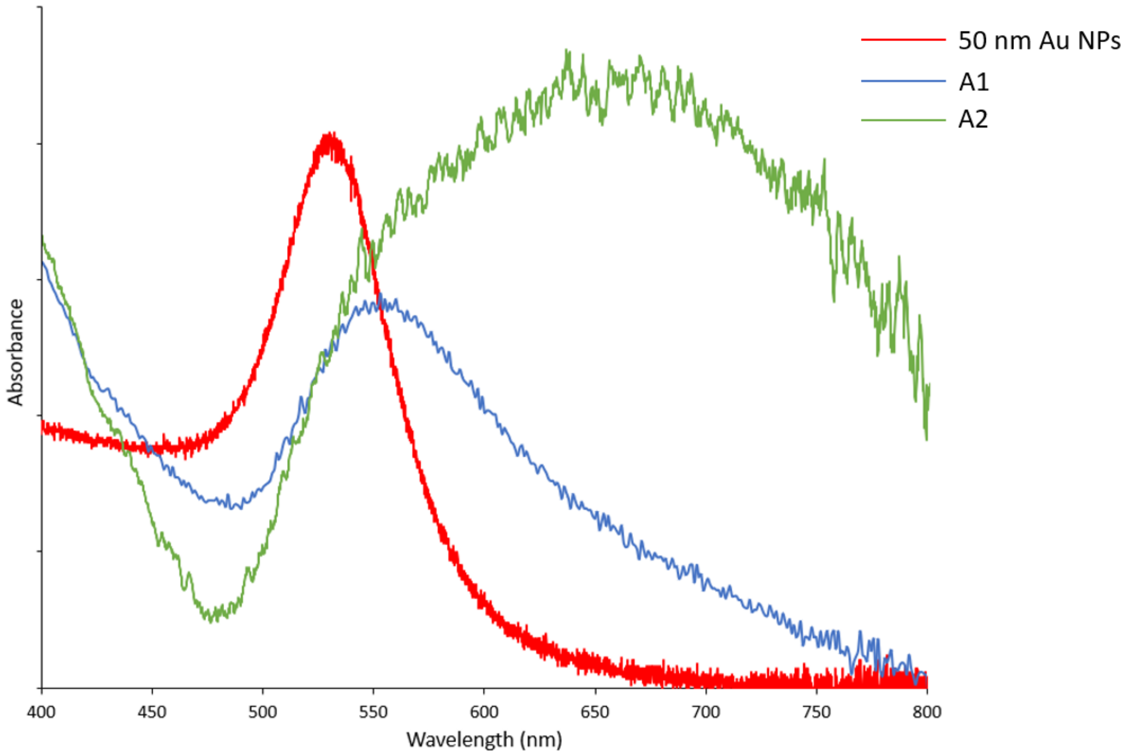

Figure S4: UV-Vis data of 50 nm gold colloid solution and experiments A1 and A2

1.2 Supplementary Tables

Table S1:Electrode potentials and half equations of different elements (Milazzo et al., 1978)

Element	Half equation	E^o / V
Fe	$Fe^{2+}(aq) + 2e^{-} \rightleftharpoons Fe(s)$	-0.440
Co	$Co^{2+}(aq) + 2e^{-} \rightleftharpoons Co(s)$	-0.277
Ni	$Ni^{2+}(aq) + 2e^{-} \rightleftharpoons Ni(s)$	-0.257
Fe	$Fe^{3+}(aq) + 3e^{-} \rightleftharpoons Fe(s)$	-0.037
H_2	$2H^{+}(aq) + 2e^{-} \rightleftharpoons H_2(g)$	0.000
Ag	$Ag^{+}(aq) + e^{-} \rightleftharpoons Ag(s)$	0.780
Au	$AuCl_4^{-} + 3e^{-} \rightleftharpoons Au(s) + 4Cl^{-}$	1.002

Table S2: XPS characterisation with different iron sources

Iron Source	Atomic Concentration %			
	Fe	B	Na	Fe/B
$FeCl_2$	10.8	3.5	7.3	3.13
$FeCl_3$	6.7	6.7	3.2	1.10

Table S3: Reaction conditions used for the experiments of Fe-B@Au

Exp	A1	A2
Addition rate of gold precursor solution	25 ml/hr	Rapidly
Temperature of gold precursor solution (^oC)	19	4
Temperature of core particle dispersion (^oC)	60	125
Colour of mixture	Burgundy	Black/blue
Core-Shell formed?	No	Partial

Table S4: ICP analysis of Exp A2

Element	Molar ppm	% split in whole particle	Fe/B
B	1.20	39.87	0.64
Fe	0.76	25.25	
Au	1.05	34.88	-
